# Negative Body Attitudes and Sexual Dissatisfaction in Men: The Mediating Role of Body Self-Consciousness During Physical Intimacy

**DOI:** 10.1007/s10508-017-1016-3

**Published:** 2017-06-23

**Authors:** Femke van den Brink, Manja Vollmann, Lot C. Sternheim, Lotte J. Berkhout, Renée A. Zomerdijk, Liesbeth Woertman

**Affiliations:** 1 Department of Clinical Psychology, Utrecht University, P.O. Box 80140, 3508 TC Utrecht, The Netherlands; 2 Department of Health Psychology, University of Hagen, Hagen, Germany

**Keywords:** Body image, Sexual dissatisfaction, Objectification theory, Muscularity

## Abstract

Previous research indicated that negative attitudes about the body and appearance are common among men and demonstrated that negative body attitudes are associated with negative sexual experiences. The present study investigated the association between body attitudes and sexual dissatisfaction and the mediating role of body self-consciousness during physical intimacy. In a cross-sectional design, 201 Dutch men completed an online survey regarding body attitudes toward muscularity, body fat, height, and genitals, body self-consciousness during physical intimacy, and sexual dissatisfaction. Hypotheses were tested using correlation analyses and a mediation analysis with body attitudes as predictors, body self-consciousness as mediator, and sexual dissatisfaction as outcome. Correlation analyses showed that negative body attitudes and body self-consciousness during physical intimacy were significantly related to sexual dissatisfaction. The mediation analysis revealed that negative attitudes toward muscularity, body fat, and genitals had indirect effects on sexual dissatisfaction through body self-consciousness during physical intimacy. Negative attitudes toward genitals additionally had a direct effect on sexual dissatisfaction. These findings indicate that body image interventions focused on male body attitudes may be beneficial in improving men’s body image, which may ultimately increase sexual satisfaction.

## Introduction

For most individuals, pleasurable sexual experiences are an essential element of overall health-related quality of life (e.g., Henderson, Lehavot, & Simoni, 2009; Robinson & Molzahn, 2007). Research has indicated that sexual dissatisfaction is associated with lower quality of life and well-being (Heiman, 2002; Nicolosi, Moreira, Villa, & Glasser, 2004; Tan, Tong, & Ho, 2012). Published studies on this topic suggest that 15–41% of men are dissatisfied with their sex life (Dunn, Croft, & Hackett, 2000; Frederick, Lever, Gillespie, & Garcia, 2017; Mulhall, King, Glina, & Hvidsten, 2008; Pedersen & Blekesaune, 2003). Since sexual dissatisfaction can affect overall quality of life, identifying determinants thereof is important. To this end, we examined links between male body image and sexual dissatisfaction.

Body image is a multidimensional construct, but research has mainly focused on the attitudinal-evaluative component (Cash, 2002). Previous studies of men have indicated that negative attitudes about the body and appearance are common (Frederick & Essayli, 2016; Frederick, Forbes, Grigorian, & Jarcho, 2007; Frederick, Sandhu, Morse, & Swami, 2016; Griffiths et al., 2016; Ridgeway & Tylka, 2005). These findings are concerning in the context of men’s sexual experiences, since negative body attitudes were found to be associated with greater sexual dissatisfaction (Gil, 2007; Holt & Lyness, 2007; Peplau et al., 2009; Træen, Markovic, & Kvalem, 2016).

A mechanism through which negative body attitudes might be linked to sexual dissatisfaction can be found in Fredrickson and Roberts’ (1997) objectification theory. This theoretical framework was originally developed to explain women’s experiences and posits that the treatment of women as sexual objects by men and in the media leads women to seeing themselves as objects to be evaluated based upon bodily appearance (i.e., self-objectification). Self-objectification is manifested as persistent consciousness of the body and habitual body monitoring (e.g., Roberts & Gettman, 2004) and has been linked to numerous negative outcomes, such as sexual dissatisfaction (Fredrickson & Roberts, 1997). However, given the increased cultural emphasis on men’s appearance, objectification theory is now considered relevant for understanding men’s experiences as well (Frederick et al., 2007; Moradi & Huang, 2008; Strelan & Hargreaves, 2005a).

A muscular male body ideal is much more dominant in modern society than in the past (Frith & Gleeson, 2004; Pope, Phillips, & Olivardia, 2000). Although men do not typically experience sexual objectification to the same extent as women, men’s bodies are also evaluated and judged by women and other men (Strelan & Hargreaves, 2005b). Furthermore, men are exposed to media images portraying muscular men as prestigious and attractive (e.g., Frederick, Fessler, & Haselton, 2005), which may lead them to engage in self-objectification (Aubrey, 2006). Men’s self-objectification was found to predict negative body attitudes (Morry & Staska, 2001; Strelan & Hargreaves, 2005a). Particularly during physical intimacy, in which the body is exposed to a partner, negative attitudes toward one’s body may increase the likelihood of becoming more conscious about the body. Exaggerated body self-consciousness during physical intimacy may, in turn, interfere with focusing on sexual pleasure (Fredrickson & Roberts, 1997), which may contribute to sexual dissatisfaction.

Previous research supports this assumption by providing empirical evidence of the mediating role of body self-consciousness during physical intimacy in the relationship between negative body attitudes and sexual dissatisfaction. Sanchez and Kiefer (2007) found that body shame was related to greater body self-consciousness during physical intimacy, which, in turn, predicted lower sexual pleasure. This mediating role of body self-consciousness during physical intimacy was supported by findings of Penhollow and Young (2008) and Milhausen, Buchholz, Opperman, and Benson (2015), who found that body self-consciousness during physical intimacy was associated with sexual dissatisfaction in samples of young adult men. In contrast, Daniel and Bridges (2013) found no significant relationship between body self-consciousness and sexual dissatisfaction. This could be explained by the fact that, compared to the other studies, men’s general body self-consciousness (i.e., body self-consciousness without specifying a particular situational context), instead of context-specific body self-consciousness (i.e., body self-consciousness during physical intimacy), was assessed, indicating that the latter may be particularly relevant in predicting sexual dissatisfaction. In sum, empirical studies have indicated that negative body attitudes are indirectly related to sexual dissatisfaction in men through body self-consciousness during physical intimacy.

It is important to note that few instruments intending to measure men’s body attitudes have been developed (Tylka, Bergeron, & Schwartz, 2005). Commonly, studies in men use instruments originally developed to measure women’s body image. However, body image is appraised differently in men. Men generally strive for a “muscular mesomorph” body shape with muscled arms and shoulders, small waist, and low body fat (Cohane & Pope, 2001; Labre, 2005; Mishkind, Rodin, Silberstein, & Striebel-Moore, 1986). Besides muscularity and low body fat, tall height as well as evaluation of the genitals was identified as an important elements of men’s body image (Frederick, Peplau, & Lever, 2006; Morrison, Bearden, Ellis, & Harriman, 2005; Gaither et al., 2016; Ridgeway & Tylka, 2005; Tiggemann, Martins, & Churchett, 2008). The body image measures used in previous studies investigating the relationship between body attitudes and sexual dissatisfaction in men do not (fully) cover these important aspects of male body image. Including unique aspects of male body attitudes in research may provide more complete and accurate results.

To summarize, objectification theory and results of empirical studies suggest that negative body attitudes are meaningfully linked to sexual dissatisfaction in men. The mediating role of body self-consciousness during physical intimacy in this relationship may be particularly salient. However, given the lack of sufficient male body image measures (i.e., measures including unique aspects of male body attitudes) in prior research, previous findings may not present the full picture of the specific negative body attitudes related to sexual dissatisfaction. Since male body image is multifaceted (Tiggemann et al., 2008) and today’s men experience increased societal and media pressures to meet an unrealistic body ideal (Pope et al., 2000), further research focusing on identifying and targeting unique aspects of men’s body image concerns and the sexual problems that can accompany these concerns would be valuable.

### The Present Study

**Fig. 1:**
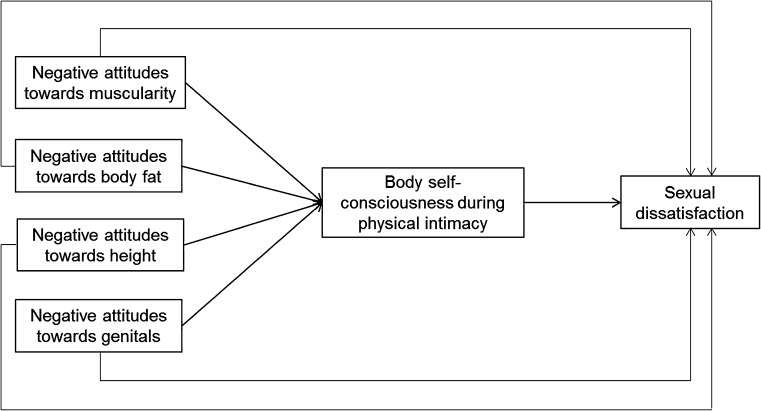
Schematic summary of the hypothesized links between body attitudes with sexual dissatisfaction and the mediating role of body self-consciousness during physical intimacy

The present study investigated the relationships between body attitudes, body self-consciousness during physical intimacy, and sexual dissatisfaction in men. On the basis of previous findings regarding the unique aspects of male body image, we focused on body attitudes toward muscularity, body fat, height, and genitals. Based on the associations between negative body attitudes, body self-consciousness during physical intimacy, and sexual dissatisfaction found in previous studies (e.g., Holt & Lyness, 2007; Milhausen et al., 2015), it was expected that negative attitudes toward muscularity, body fat, height, and genitals as well as higher levels of body self-consciousness during physical intimacy would be associated with greater sexual dissatisfaction. Additionally, based on previous findings (e.g., Sanchez & Kiefer, 2007), it was expected that body self-consciousness during physical intimacy would mediate the relationships between the aspects of body attitudes and sexual dissatisfaction. These proposed hypotheses are summarized schematically in Fig. 1.

## Method

### Participants and Procedure

Participants were recruited from a university community via posters displayed in the social sciences department, flyers distributed on campus, and the student Web site of Utrecht University which provides an overview of all ongoing research projects. Heterosexual men that are or have been sexually active with a female partner were invited to take part in an online study on “Body Image and Sexual Satisfaction in Men.” A short description of the study and the direct link to the online questionnaire were given. Interested men could access the questionnaire via that link. After opening the link, participants had to complete an informed consent form, in which voluntary participation and anonymity were highlighted. In order to avoid missing data, all questions were mandatory. Social sciences students from Utrecht University received course credit for participation. All other participants were not compensated for participation. On average, it took 30 min to complete the questionnaire.

Sample size calculations (Faul, Erdfelder, Lang, & Buchner, 2007; Fritz & MacKinnon, 2007) revealed that 177 participants would be required in order to detect small to medium effects (under guidelines from Cohen, 1988, p. 412) with 80% power and a type I error rate of 5%. The estimated effect size was based on effects found in similar past research (Holt & Lyness, 2007; Sanchez & Kiefer, 2007; Træen et al., 2016).

A total of 201 men fully completed the questionnaire. Participants’ age ranged from 18 to 44 years with a mean age of 23.88 (SD = 4.23). A total of 69 men (34.4%) received course credit for participation. The majority of participants (67.1%, *n* = 135) had a romantic partner. The duration of the romantic relationship was less than 1 month in 5.5% (*n* = 11), between 1 and 6 months in 11.6% (*n* = 23), between 6 and 12 months in 9.5% (*n* = 19), between 1 and 2 years in 10.4% (*n* = 21), and longer than 2 years in 32.4% (*n* = 65) of these participants. Highest level of education (completed or current) was lower secondary school in 7.5% (*n* = 15), higher secondary school in 22.9% (*n* = 46), lower vocational education in 7.0% (*n* = 14), higher vocational education in 19.9% (*n* = 40), and university in 42.8% (*n* = 86) of the participants.

### Measures

All scales were translated from English to Dutch using the translate–retranslate method (retranslation by a native speaker), unless otherwise stated. Means, SDs, and minimum and maximum scores for each of the measures are shown in Table 1.

**Table 1:** Means, SDs, minimum and maximum scores, and bivariate correlations between the aspects of body attitudes, body self-consciousness during physical intimacy, and sexual dissatisfaction

	*M*	SD	Minimum	Maximum	1	2	3	4	5
1. Negative attitudes toward muscularity^a^	2.43	.86	1.00	6.00	–	–	–	–	–
2. Negative attitudes toward body fat^a^	2.36	.91	1.00	5.88	−.03	–	–	–	–
3. Negative attitudes toward height^a^	2.28	1.29	1.00	6.00	.20***	.12	–	–	–
4. Negative attitudes toward genitals^b^	1.89	.50	1.00	3.86	.17*	.26***	.21***	–	–
5. Body self-consciousness during physical intimacy^c^	1.50	.55	1.00	3.71	.37***	.36***	.24***	.56***	–
6. Sexual dissatisfaction^c^	1.80	.40	1.10	3.40	.15*	.16*	.16*	.44***	.53***

*** *p* < .001; * *p* < .05

^a^Scale range: 1–6 with higher scores indicating more negative attitudes

^b^Scale range: 1–4 with higher scores indicating more negative attitudes

^c^Scale range: 1–5 with higher scores indicating more body self-consciousness during physical intimacy/sexual dissatisfaction

#### Body Attitudes

The three subscales of the Male Body Attitudes Scale (MBAS) (Tylka et al., 2005) were used to assess body attitudes with respect to muscularity (10 items, e.g., “I think I have too little muscle on my body”), body fat (8 items, e.g., “I am concerned that my stomach is too flabby”), and height (2 items, e.g., “I wish I were taller”). The items were answered on a 6-point Likert scale ranging from 1 = never to 6 = always. Items were recoded if appropriate and averaged so that higher subscale scores indicated more negative attitudes with respect to muscularity, body fat, and height, respectively. Previous research supported the measure’s scale score reliability, and construct, concurrent, and discriminant validity (Tylka et al., 2005). Cronbach’s alpha in the current study was .90, 95% CI [.88, .92], for both the muscularity and body fat subscale, and .84, 95% CI [.79–.88], for the height subscale.

Additionally, the 7-item Male Genital Self-Image Scale (MGSIS) (Herbenick, Schick, Reece, Sanders & Fortenberry, 2013) was used to assess body attitudes with respect to the genitals (e.g., “I am satisfied with the size of my genitals”). The items were answered on a 4-point Likert scale ranging from 1 = strongly disagree to 4 = strongly agree. Items were averaged with higher scores indicating more negative attitudes with respect to the genitals. Herbenick et al. reported high-scale score reliability and good construct and discriminant validity. In the current sample, Cronbach’s alpha was .85, 95% CI [.82–.88].

#### Body Self-Consciousness During Physical Intimacy

The 17-item Male Body Image Self-Consciousness Scale (M-BISC; McDonagh, Morrison & McGuire, 2010) was used to measure body self-consciousness during physical intimacy (e.g., “During sex, I would worry that my partner would think my chest is not muscular enough”). The items were answered on a 5-point Likert scale from 1 = strongly disagree to 5 = strongly agree. Items were averaged with higher scores indicating higher body self-consciousness during physical intimacy. Research has supported the reliability and psychometric validity of the M-BISC (McDonagh et al., 2010). Cronbach’s alpha of the current study was .94, 95% CI [.93–.95].

#### Sexual Dissatisfaction

The Dutch version (Ter Kuile, Lankveld, Kalkhoven, & van Egmond, 1999) of the 28-item male version of the Golombok Rust Inventory of Sexual Satisfaction (GRISS; Rust & Golombok, 1986) was used to measure sexual dissatisfaction (e.g., “Do you feel there is a lack of love and affection in your sexual relationship with your partner?”). Items were scored on a 5-point Likert scale from 1 = always to 5 = never. Items were recoded if appropriate and averaged so that higher scores indicate greater sexual dissatisfaction. Previous research indicated good scale score reliability and validity (Ter Kuile et al., 1999). Cronbach’s alpha in the current study was .87, 95% CI [.84–.89].

### Statistical Analysis

All statistical analyses were performed with IBM SPSS Statistics version 24. In a first step, bivariate associations between the study variables were analyzed using Pearson correlation coefficients. In a second step, a mediation analysis with the four aspects of body attitudes (i.e., negative body attitudes toward muscularity, body fat, height, and genitals) as independent variables, body self-consciousness during physical intimacy as mediator, and sexual dissatisfaction as dependent variable was conducted. As previous research has shown that men involved in romantic relationships were significantly more likely to be sexually satisfied than men who were not involved in such relationships (e.g., Higgins, Mullinax, Trussell, Davidson, & Moore, 2011), relationship status was entered as control variable. The mediation analysis comprised a number of subanalyses that estimated the total, direct, and indirect effects of the four aspects of body attitudes on sexual dissatisfaction. The total and direct effects were estimated by means of a stepwise multiple regression analysis in which the four aspects of body attitudes were entered in the first step and body self-consciousness during physical intimacy was entered in the second step. Total effects refer to the specific relationships between each aspect of body attitudes and sexual dissatisfaction while controlling for the other aspects of body attitudes (first step), and direct effects refer to the specific relationships between each aspect of body attitudes and sexual dissatisfaction while controlling for the other aspects of body attitudes and body self-consciousness during physical intimacy (second step).

As recommended by Hayes (2013), the specific indirect effects of the four aspects of body attitudes on sexual dissatisfaction through body self-consciousness during physical intimacy and their significance were determined by means of bootstrap analyses with 5000 bootstrap samples and bias corrected and accelerated 95% confidence intervals (BCa 95% CI). To this end, the PROCESS macro for SPSS has been used (Hayes, 2013). All coefficients will be reported in standardized form.

## Results

### Bivariate Associations Between the Aspects of Body Attitudes, Body Self-Consciousness During Physical Intimacy, and Sexual Dissatisfaction

The results of the correlation analyses of the study variables are shown in Table 1. As expected, negative body attitudes toward muscularity, body fat, height, and genitals as well as body self-consciousness during physical intimacy were significantly related to greater sexual dissatisfaction. Also, negative body attitudes toward muscularity, body fat, height, and genitals were related to higher body self-consciousness during physical intimacy.

### Total, Direct, and Indirect Effects Through Body Self-Consciousness During Physical Intimacy of Body Attitudes on Sexual Dissatisfaction

The assumptions of multiple regression analysis (i.e., normality, linearity, homoscedasticity) were tested, and all were met. The results are shown in Table 2. A significant total effect (Step 1) and a significant direct effect (Step 2) of negative body attitudes toward genitals on sexual dissatisfaction were found. This indicates that more negative attitudes toward genitals were related to greater sexual dissatisfaction. Additionally, the analysis revealed a significant direct effect (Step 2) of body self-consciousness during physical intimacy on sexual dissatisfaction, indicating that more body self-consciousness during physical intimacy was associated with greater sexual dissatisfaction. Thirty-one percent of the variance in sexual dissatisfaction could be explained.

**Table 2:** Results of the stepwise regression analysis with sexual dissatisfaction as outcome: total and direct effects of four aspects of body attitudes on sexual dissatisfaction

Predictors	*β* Step 1	*β* Step 2
Step 1: adj. *R* ^2^ = .21, *F*(5, 195) = 11.70***		
Negative attitudes toward muscularity	.05	−.07
Negative attitudes toward body fat	.05	−.06
Negative attitudes toward height	.06	.03
Negative attitudes toward genitals	.38***	.20**
Control variable relationship status^a^	−.16*	−.11
Step 2: Δ*R* ^2^ = .10, *F*(1,194) = 28.53***; adj. *R* ^2^ = .31, *F*(6, 194) = 15.88***		
Body self-consciousness during physical intimacy		.43***

*β*s in Step 1 represent total effects of the body attitudes on sexual dissatisfaction. *β*s in Step 2 represent direct effects of the body attitudes on sexual dissatisfaction

*** *p* < .001, ** *p* < .01, * *p* < .05

^a^0 = no romantic partner, 1 = romantic partner

The bootstrap analyses revealed significant indirect effects of negative attitudes toward muscularity, .13, BCa 95% CI [.060, .223], negative attitudes toward fat, .12, BCa 95% CI [.051, .221], as well as negative attitudes toward genitals, .17, BCa 95% CI [.086, .276], on sexual dissatisfaction via body self-consciousness during physical intimacy. Thus, as expected, more negative attitudes toward muscularity, body fat, and genitals were related to higher body self-consciousness during physical intimacy, which, in turn, was related to greater sexual dissatisfaction. No significant indirect effect of negative attitudes toward height on sexual dissatisfaction via body self-consciousness during physical intimacy was found, .03, BCa 95% CI [−.019, .095].

## Discussion

The present study investigated associations of four key aspects of male body attitudes (muscularity, body fat, height, and genitals) and body self-consciousness during physical intimacy with sexual dissatisfaction. As expected and in line with previous studies (e.g., Træen et al., 2016), more negative body attitudes toward muscularity, body fat, height, and genitals were all significantly related to greater sexual dissatisfaction. The present study expanded previous research on body attitudes and sexual dissatisfaction in men by incorporating unique aspects of male body image, instead of using global, non-gender specific body attitudes measures.

The results revealed that, when considering all body attitudes simultaneously, only negative attitudes toward genitals were significantly related to greater dissatisfaction. This, however, is not surprising since genitals play a prominent role in many sexual acts (e.g., intercourse) and therefore naturally more salient in sexually intimate situations than muscularity, body fat, and body height. This finding may contribute to a better understanding of the link between negative body attitudes and sexual dissatisfaction found in previous studies. It can be speculated that the relationship between negative general body attitudes and sexual dissatisfaction particularly results from negative attitudes toward genitals. Attitudes toward genitals may affect men’s more general views of their bodies, creating insecurity for men who are dissatisfied with their genitals and confidence for men who are satisfied with their genitals (Lever, Frederick, & Peplau, 2006). These findings underline the importance of attitudes toward genitals in the conceptualization of male body image (Tiggemann et al., 2008).

Our results further showed, as expected and in line with objectification theory and previous findings (e.g., Fredrickson & Roberts, 1997; Penhollow & Young, 2008), a statistically significant association between body self-consciousness during physical intimacy and sexual dissatisfaction. Thus, during physically intimate interactions with a partner, where the body is unavoidably at focus, an increase in body self-consciousness may disrupt men’s sexual satisfaction.

Most importantly, this study offers further insight into the role of body self-consciousness during physical intimacy in the association between negative body attitudes and sexual dissatisfaction. The results of the mediation analysis suggest that negative body attitudes toward muscularity, body fat, and genitals may activate body self-consciousness in sexually intimate situations, which, in turn, leads to greater sexual dissatisfaction. Body attitudes toward height were not related to sexual dissatisfaction, suggesting that negative attitudes about those aspects of the body that become more apparent for a partner during physical intimacy may have an impact on sexual dissatisfaction.

Thus, men’s concerns about parts of their bodies that might have their origin in an inflated cultural male body ideal (e.g., Labre, 2005) are likely to manifest themselves in the form of exaggerated body self-consciousness during physical intimacy with a partner that hinders focusing on sexual pleasure and positive sexual experiences (Fredrickson & Roberts, 1997).

Negative attitudes toward genitals were also directly related to sexual dissatisfaction, again highlighting the importance of this aspect of male body image in the context of sexuality. Previous research indicated that negative body attitudes are associated with lower sexual esteem (i.e., an individual’s confidence in themselves as a sexual partner; Wiederman & Allgeier, 1993) (Morrison et al., 2005) and sexual avoidance (La Roque & Cioe, 2011). Since many men perceive that the size of their penis is closely associated with masculinity and sexual performance (Francken, Van de Wiel, Van Driel, & Schultz, 2002; Lever et al., 2006), negative attitudes toward genital appearance may translate into feelings of insecurity about sexual competence, which may lead to avoidance of sexual activity with a partner and not experiencing the satisfaction normally associated with physical intimacy. However, this explanation remains hypothetical needing further investigation.

The results of this study may have potential implications for clinical practice. For therapists who treat clients with sexual problems, body image concerns may not be easily identified because men tend to avoid discussing these concerns with others (e.g., Barwick, Bazzini, Martz, Rocheleau, & Curtin, 2012). Besides, body image is often not been seen as a men’s issue (Tantleff-Dunn, Barnes, & Larose, 2011) and men may be therefore unlikely to disclose distress related to feelings about the appearance of their body. It is therefore important that therapists pay attention to potential body image issues and should address these issues with male clients if needed. Incorporating body image intervention would be useful in this respect. For example, cognitive-behavioral body image therapy has been found to be an efficacious treatment of body image problems (for a meta-analysis, see Jarry & Ip, 2005), with outcomes shown to reduce body self-consciousness during physical intimacy (Grant & Cash, 1996). This form of intervention may be beneficial for improving men’s body image, which in turn can result in positive sexuality outcomes.

In addition, this study adds to existing literature by offering more insight into the unique aspects of male body image important in the context of sexual dissatisfaction. Male body image is multifaceted and, as highlighted by Tiggemann et al. (2008), “male body image cannot be adequately conceptualized and studied by simply tweaking our previous investigations of female body image” (p. 1168). As today’s men experience increased societal and media pressures to meet an unrealistic body ideal (Pope et al., 2000), it will become increasingly important to identify and target unique aspects of men’s body image concerns, and the sexual problems that can accompany these concerns.

### Limitations

Some limitations need to be acknowledged. The present sample consisted of heterosexual and primarily highly educated young men. Because of the homogeneous sample, results of this study may not be representative of the general Dutch population of men. Future research would profit from more heterogeneous samples and from including bisexual and gay men while taking specific aspects of same-sex sexuality into account (McDonagh, Stewart, Morrison, & Morrison, 2016; Sandfort & de Keizer, 2001).

Furthermore, a measure of body mass index (BMI) was not included in this study, which in retrospect was an oversight as BMI has been associated with body attitudes and with sexual experiences (e.g., Frederick & Jenkins, 2015). In addition, in the present study, within-person effects have been investigated. As sexual relationships are dyadic in nature, perceptions and behaviors of the sexual partner might be of importance with regard to the quality of sexual experience (Zhaoyang & Cooper, 2013). Further studies should include data on BMI and use dyadic designs to take the interdependence of partners into account.

Lastly, given the cross-sectional nature of this study, direction of causality could not definitely be determined. Although mediation analyses are common statistical procedures on cross-sectional data, further longitudinal studies are needed to study the effects over time (Maxwell, Cole, & Mitchell, 2011).

### Conclusions

Despite the limitations, this study adds to the literature by targeting relationships between body image and sexual experiences in men. Given the fact that cultural body ideals for men have become unattainably masculine in the past decades (Pope et al., 2000), it is important to further study potential health risks of males related to their body image. Understanding how different aspects of body image in men relate to sexual dissatisfaction will be valuable in selecting the appropriate targets for treatment intervention in the context of body image issues and sexual problems.
